# The integrated analysis of primary and secondary incident characteristics: Focusing on the impact and scope of the safety service patrol program in Iowa

**DOI:** 10.1016/j.heliyon.2023.e17759

**Published:** 2023-06-28

**Authors:** Minsoo Oh, Jonathan Wood, Jing Dong-O’Brien

**Affiliations:** Department of Civil, Construction and Environmental Engineering, Iowa State University, Ames, IA, 50011, USA

**Keywords:** Binary logistic regression, Primary incident, Safety service patrol program, Secondary incident, Survival analysis

## Abstract

Secondary incidents are considered a major risk in terms of traffic management due to dangerous ramifications, such as reduced capacity, additional traffic delays, and serious injuries. Therefore, it is necessary to examine what causes a secondary incident to occur after a primary incident and prepare countermeasures to reduce the possible damage to human and property from primary and secondary incidents. In Iowa, a safety service patrol program is being implemented on major highway routes to respond to both types of incident efficiently. However, research on when, where, and under what conditions these incidents occur and how the program can deal with incidents must be conducted to determine the major characteristics of primary and secondary incidents and to estimate the program's performance. Consequently, statistical and spatial analyzes were performed on traffic incidents in a 5-year period (2016–2020) in Iowa. A survival analysis confirmed that the program could decrease the probability of secondary incident occurrences, and 99.9% of secondary incidents occurred within 4 h of the primary incident. Additionally, the binary logistic regression analysis of primary incidents affirmed that a longer incident clearance time and a higher severity of incidents could increase the probability of secondary incidents occurrence. Furthermore, a spatial analysis evaluated that the Iowa DOT safety service patrol program adequately covered areas where primary and secondary incidents are focused. This study is expected to be used to develop countermeasures in both incident cases by identifying the characteristics of secondary and primary incidents in Iowa.

## 1 Introduction

Vehicle incidents on highways are often more severe than incidents that occur on other road types due to high speeds and heavy traffic volumes. Moreover, highway incidents, such as vehicle crashes, negatively impact traffic flows, resulting in increased travel delays and secondary incidents. Therefore, traffic operations and management agents develop policies to manage incidents on highways [[1], [2], [3]]. For instance, the Iowa Department of Transportation (DOT) introduced a safety service patrol (SSP) program called Highway Helper (HH) in major metropolitan areas in 2015. The program provides travelers and emergency responders with free assistance, including starting a battery, changing tires, 2 gallons of fuel, transport to a safe location, traffic control, and sweeping or moving debris from the roadway. Currently, Iowa's Department of Transportation operates the program in Des Moines, Council Bluffs, Iowa City-Cedar Rapids, and the Quad Cities. Benefit-cost analyses of the operation of the program in the areas that were found to be cost-effective in the three regions, except for the Quad Cities area, where the program was first available in 2019 [4].

Existing research on the safety benefits of the Iowa service patrol program, focusing on the management of primary and secondary incidents, is insufficient. Furthermore, the severity of primary and secondary incidents that occurred within Iowa has not been addressed in previous studies. This study includes an analysis of the characteristics of primary incidents that are associated with an increased risk of secondary incidents. Therefore, further investigation of which characteristics of primary incidents are related to the occurrence of secondary incidents should be performed, including the program's benefits in managing primary and secondary incidents. These characteristics should be systematically analyzed by scrutinizing the program's performance in terms of safety and road operations rather than a simple cost-based analysis. Therefore, this study presents comprehensive statistical analyzes of primary and secondary incidents in Iowa. This analysis was conducted to determine the characteristics of primary and secondary incidents and to determine the performance of the HH program in managing both types of incidents. Furthermore, based on the primary and secondary incidents that occurred between 2016 and 2020 (as recorded by the responding agencies), spatial investigations are used to determine whether the routes of the SSP program can reduce the risk of secondary incidents in the operating regions of the HH in Iowa.

This paper is organized as follows: First, in the literature review, the existing literature on the analysis of primary and secondary incidents is reviewed and illustrates the trend of research related to SSP programs. The elements required for statistical and spatial analyzes are also identified and the justifications for developing the methods used for this research, based on gaps in the literature, are presented. The methodology section explains the statistical techniques used in this study, Survival Analysis and Binary Logistic regression, and introduces the spatial analysis procedure. The Data Collection and Description Section provides the incident descriptive statistics and the other data sources used. In addition, the results of a T-test on how the SSP can benefit from managing primary and secondary incidents are introduced. Lastly, the results section is divided into two parts: 1) primary and secondary incidents, and 2) spatial analysis. The primary and secondary incident analysis describes which factors of primary incidents are related to the occurrence of secondary incidents. In the spatial analysis section, an evaluation was conducted that compares the SSP program and the outcomes of traffic safety.

## 2 Literature review

The main topics covered in this study are primary and secondary incident statistical analysis and the evaluation of the suitability of the SSP program implementation in Iowa, USA. A secondary incident can be defined as an incident that occurs within a specific spatial and temporal range after a primary incident (which leads to the secondary incident occurring) [5].

Previous studies related to secondary incidents have been conducted with various objectives, including secondary incident identification, secondary incident prediction, and secondary incident management. Various approaches have been used to identify secondary incidents, such as spatial-temporal range-based methods [[6], [7], [8], [9], [10]], queuing model-based methods [[11], [12], [13], [14], [15], [16], [17]], speed contour map-based methods [[18], [19], [20], [21], [22]], and shockwave-based methods [15,[23], [24], [25]], have been performed. Although there is research in each of these areas, further research is still needed in each of these areas and an improved understanding of the characteristics of secondary incidents [26]. Furthermore, since the incident data used in this study includes identifiers for secondary incidents, this review of the literature focuses on the aspects related to the prediction and prevention of secondary incidents rather than on identification.

Several studies have been conducted to determine the factors that cause secondary crashes. One of these studies estimated a logit model to estimate the relationship between the occurrence of a secondary incident and the clearance time of the primary incident. The study found that vehicle location, vehicle type, day of week, and season are significant factors in increasing secondary crash likelihood [6]. Furthermore, this study estimated a binary logit model to estimate the likelihood of secondary collision, which showed that the likelihood of secondary collision increased with longer lane blockage durations, resulting in longer queues and congestion. The authors explained that secondary incidents could occur more during specific periods, such as peaks in the morning and afternoon peaks and during the midday hours. Furthermore, several factors, including time of day, number of vehicles, type of incident, duration of lane blockage, and number of lanes, were shown to be associated with secondary crashes [8,12]. However, some studies argued that factors that explain the probability of a secondary crash, based on time changes, are not considered in the design and interpretation of models [27,28]. The prediction models include highly correlated variables, so multicollinearity issues are probably inherent in the models. One of the studies pointed out that there are two significant issues for prediction modeling analysis. First, most of the models' explanatory variables are considered highly correlated. Second, the proportion of primary and secondary incidents is a small part of all incidents, resulting in an unbalanced classification problem (i.e., sample size imbalances) [27]. Lastly, a study developed a high-order Markov model to predict the likelihood of secondary crashes reflecting the duration of the traffic accident. The study insisted that the statistical models of previous studies that estimate the probability of secondary crashes did not take into account changes in probability [28].

Several states in the United States have implemented SSP programs to mitigate traffic congestion and reduce secondary incidents. Although the name of the program varies by state, its main purpose is to minimize the consequences of highway incidents in terms of highway operation and safety perspectives by responding quickly responding to and managing incidents. Consequently, previous studies have focused on developing benefit evaluation methodologies for SSP programs. The performance measures of the SSP programs - reductions in incident clearance time, roadway clearance time, number of secondary crashes, and incident-induced delay - were the main outcomes evaluated in previous research [[29], [30], [31]]. In particular, the core of the evaluation of the program is the accurate calculation of incident-induced delay, so many studies on deterministic queuing theory have been conducted focusing on data types or fine-tuning the parameters of the theory [1,[32], [33], [34], [35], [36], [37]].

In summary, explanatory variables for a secondary incident predictive model and expected issues are derived from the existing literature review related to the analysis of primary and secondary incidents. Explanatory variables selected in the secondary incident prediction models were mainly the clearance time of the primary incidents, the type of vehicle, the duration of the lane block and the number of vehicles. The main incident factors that are found to be related to the occurrence of secondary incidents are used as explanatory variables in this article. Furthermore, issues regarding sample size imbalance and time series are emphasized among the expected issues for analyzing primary and secondary incidents. In this study, a general incident is defined as an incident that does not fall into the category of primary and secondary incidents, and the primary incident is an incident that causes at least one secondary incident. The sample size between general, primary, and secondary incidents was imbalanced. Additionally, most studies did not consider the time flow from the occurrence of a primary incident. Therefore, this research used both survival analysis and binary logistic regression to overcome the limitations of the statistical models used in previous studies. Finally, research on how the SSP program can benefit from managing primary and secondary incidents and whether the SSP program covers the regions where primary and secondary incidents occur frequently in the state is insufficient. Accordingly, this research introduces an approach to determine whether the state-level SSP program covers regions concentrated by the occurrences of both primary and secondary incidents in Iowa.

## 3 Methodology

**Fig. 1:**
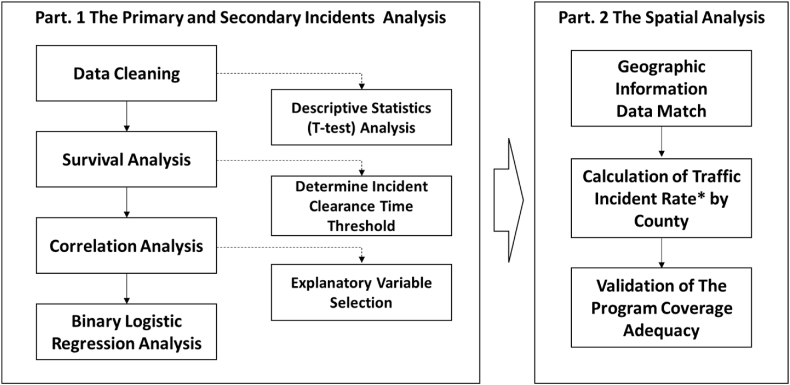
Research framework.

The methodology of this study consists of two parts: the primary and secondary incident analysis and the SSP program coverage evaluation (Spatial Analysis). Primary and secondary incident analysis consists of four processes: Data cleaning, survival analysis, correlation analysis, and binary logistic regression (BLR) analysis. The spatial analysis of the program is divided into three parts: Geo-information match, Calculation of traffic incident rate, and Validation of the program coverage adequacy (Fig. 1).

To begin with, data cleaning was performed. In the entire incident dataset, cases in which incident clearance time exceeded 24 h were considered outliers. On the basis of the clearance time dataset, descriptive statistics for each event type were calculated. In addition, incident clearance and lane blocking times are established as a measure of effectiveness to determine how the program affected primary and secondary incident management using a *t*-test. Survival analyzes were performed to decipher the relationship between the occurrence of secondary incidents and the change in time after the primary incidents.

Survival analysis has been used in multiple academic fields. Survival analysis is appropriate in this investigation because secondary incident data are uncensored, considering the characteristics that the reported incident time and incident clearance time are known and reported. The survival analysis used in the analysis is an intuitive method that shows the probability of survival over time based on the observed times of cases where an event occurred and cases where an event did not occur. The duration threshold for the incident was set based on the result of the survival analysis to increase the accuracy of the primary incident prediction model. Survival analysis was performed using the Kaplan-Meier estimation method, which is a nonparametric survival function estimation method developed by Edward L. Kaplan and Paul Meier. The surviving probability ($S_{t})$ at time t is defined as the ratio of the number of subjects that survive in a given time duration divided by the number of subjects that survive at the beginning time, which formula is defined as Equation (1) [38]. In this study, the survival probability of each time step was accumulated to produce a cumulative distribution graph, which was used to set the duration threshold for the incident.

(1) $$S_{t}=\frac{Number\;of\;subjects\;living\;at\;the\;start-Number\;of\;died}{Number\;of\;subjects\;living\;at\;the\;start}$$

Incident cases that exceeded the threshold were filtered on the basis of the incident duration threshold. The correlation analysis was then applied to determine explanatory variables that are highly related to the occurrence of secondary incidents. Using the explanatory variables derived from the correlation analysis, a binary logistic regression analysis was conducted to estimate the probability of secondary incidents. A binary logistic regression model is used to estimate the probability of occurrence of an event using linear combinations of independent variables. The dependent variable of binary logistic regression can be divided into two outcomes, success or failure. The sum of the ratio of each variable is 1, which means a probability allocated to each category. The two characteristics of binary logistic regression are as follows: First, the range of the dependent variable is restricted to the range of 0 to 1. The distribution of conditional probability follows a binomial distribution instead of a normal distribution because the dependent variable is binary. The binary logistic regression model has two commonly used primary concepts: Odds ratio and logistic function. The odds ratio is the change in the dependent variable associated with changing an independent variable when the other independent variables are constant (the odds ratio is the exponent of the estimated coefficient for the specified independent variable). The binary logistic regression form is presented in Equation (2) [39].

(2) $$P\left(Y=1|\beta \right)=\frac{e^{\beta }}{1+e^{\beta }},\beta =\beta_{0}+\beta_{1}x_{1}+\cdots +\beta_{k}x_{k}$$

For spatial analysis, an approach using a geographic information system (GIS) is devised to analyze the regions where primary and secondary incidents occurred and investigate how well the Iowa DOT SSP program covers those locations. Initially, a basic regional level for the analysis was determined as county, and the entire incident rate and the incident rate for primary and secondary incidents were calculated for each county by matching the incident data and the GIS information collected from the Iowa DOT open data portal. Incident rates were calculated as incidents per hundred million vehicle miles traveled, which is presented as Equation (3). The total number of incidents for the study period per 100 million vehicle miles traveled is computed to compare incident rates. In the case of analyzing primary and secondary incidents, it is calculated using the number of primary and secondary incidents as an incident value. After sorted by incident rate, the coverage area of the program is compared to the regions to verify the suitability of the program. Incident rates were used for comparison due to the small number of areas available for analysis (more advanced analyzes, such as count regression models, were not possible due to the limited sample size). Therefore, a linear relationship between the number of incidents and the number of miles traveled by vehicles is an inherent assumption required for spatial comparisons.

(3) $$Incident\;rate=\frac{The\;total\;number\;of\;incidents\;for\;study\;periods\times 10^{8}}{Average\;AADT\times 365\;days\times 5\;years\times Entire\;roadway\;length\;\left(miles\right)}$$

## 4 Data collection and description

For this research, statistical and spatial analyzes were performed using primary and secondary incidents in Iowa. The data required for the analysis of the primary and secondary incidents were incident information. The source of incident data is obtained from the Iowa DOT Advanced Transportation Management System (ATMS) operated by Iowa DOT. This system records information about each incident, and incident responders collect incident information primarily in the field with the support of the Traffic Management Center surveillance system, including whether the incident was primary or secondary. The incident data are a tabulated data set that includes incidents in Iowa. Incidents are aggregated by day, month, and year, creating an annualized data set. These data include detailed descriptions of incidents in Iowa and 96 variables including incident date, incident location, Severity (Injuries and Fatalities), Vehicle type, SSP dispatch, Lane blockage, secondary incident indicator, event type, reported time and cleared time. In addition, there were three data sources for spatial analyses; Iowa state highway, Iowa county, and SSP routes. The Iowa state highway information consists of about 13,000 segments, including traffic volume such as annual average daily traffic. Additionally, there are 99 counties in Iowa with specific county-level information. The owner of incident data and SSP routes is Iowa DOT. Therefore, permission was required for the incident data. For the other required data sets, Iowa's state highway and Iowa county information are open data sources, which could be obtained from the Iowa DOT open data page [40,41].

This research used incidents collected for five consecutive years from 2016 to 2020 and the primary and secondary incident types: vehicle crashes (1 vehicle, 2 vehicles, 3+ vehicles, earlier crashes), emergency vehicle, grass fire, slow traffic, stalled vehicle, towing operation and vehicle fire. Descriptive statistics are provided in Table 1.

**Table 1:** The descriptive statistics of incidents in Iowa.

Incident type		Overall	Secondary Incident	Primary Incident
Number of Incidents		150,849	635	578
Fatalities	Number of Cases	429	6	16
	Cases per 10k incidents	28.4	93.6	273.1
Injuries	Number of Cases	3,663	58	105
	Cases per 10k incidents	242.8	904.8	1791.8
Lane Blockage Time (min)	Average	7.69	19.73	41.50
	Standard Deviation	122.28	43.08	65.66
	Min	0.00	0.00	0.00
	Max	1,344.00	400.00	622.00
Clearance Time (hour)	Average	2.47	0.94	1.51
	Standard Deviation	4.22	1.63	2.25
	Min	0.02	0.02	0.05
	Max	23.98	23.82	22.42

As shown in Table 1, the total number of incidents was 150,849, resulting in 429 fatalities and 3,663 injuries. The entire incident set included 578 primary and 635 secondary incidents, resulting in 22 deaths and 163 injuries. The ratio of the primary and secondary incidents was only 0.8% of overall incidents in Iowa, but the number of fatalities and injuries was 5.1% and 4.4%, respectively. Converting the results into the number of fatalities and injuries per 10,000 incidents, the number of fatalities per 10,000 secondary incidents was about three times higher than the average of the entire incident set, and the number of injuries per 10,000 incidents was 3.73 times higher, indicating that the severity of secondary incidents is significantly higher than overall incident cases, on average. Moreover, for primary incidents that lead to secondary incidents, the number of fatalities and injuries per 10,000 incidents was three times and two times higher than for the secondary incidents, respectively.

The proportion of events due to vehicle collision was calculated as more than 80% in both primary and secondary incidents, and incidents in which at least one lane was blocked were found to be 49% and 71%, respectively. Furthermore, the descriptive statistics indicate that the lane blocking time and incident clearance times were longer for the primary incidents than for the secondary incidents. The data also indicate that the average blockage time of the lane of the primary and secondary incidents was longer than that of the general incidents, although the clearance time was shorter.

The descriptive statistics for the variables included in the binary logit model are provided in Table 2. As shown, incident clearance time ranges from 0.02 to 4 h with a mean value of 0.96 h. The number of injuries ranged from 0 to 5 with a mean value of 0.07. The number of cars ranged from 0 to 7. Vehicles involved in an incident were collected as vehicle types: Cars, SUVs, tractors and trailers, trucks, DOT trucks, and motorcycles. The number of vehicles for each type was used as an individual explanatory variable. If the vehicles involved in an incident are not in the lane or an incident is not a vehicle crash-related event: slow traffic, stalled vehicles on a shoulder or road maintenance, the total number of vehicles is marked as 0. In addition, descriptive statistics indicate that the shoulder was blocked in 54% of cases, the SSP responded to 74% of incidents, and 33% of incidents were considered primary incidents.

**Table 2:** Description of the model variables.

Type	Number	Description (Abbreviation)	Values	Statistics				
				Cases	Min	Median	Mean	Max
Continuous	1	Incident Clearance Time (ICT)	Hours	1,617	0.02	0.72	0.96	4.00
	2	Injuries (IJ)	The number of Injuries		0	0	0.07	5.00
	3	Cars (C)	The number of Cars		0	0	0.71	7.00

Type	Number	Description (Abbreviation)	Values	The number of cases	
				Yes	No
Categorical	4	Only Shoulder Blocking Indicator (SBI)	N: No Y: Yes	871 (54%)	746 (46%)
	5	Safety Service Patrol Indicator (SSPI)	0: No 1: Yes	1,203 (74%)	414 (26%)
	6	Primary Incident Indicator	0: No 1: Yes	539 (33%)	1,078 (66%)

## 5 Results

The results of the analysis conducted in this research provide information on the effectiveness of the SSP program in improving incident management and reducing the probability of secondary incidents. In this section, specific results and related discussions from survival analysis, binary logistic regression, and spatial analysis are provided and discussed.

### 5.1 Clearance and lane blockage times

Lane blockage and incident clearance times were selected as a measure of the effectiveness of the SSP program performance for the initial analysis using t-tests. Vehicle collisions that accounted for most of the majority of primary and secondary incidents were sorted so that the benefits of the SSP program were evaluated using t tests between incidents with and without the assistance of the SSP program. Among the cases that received the program's help, there were only 69 cases of two-vehicle collisions, and the number of other types of incident did not exceed 30. Therefore, it could not be assumed that the normal distribution is satisfied on the basis of the central limit theorem. Therefore, it was checked whether the variances of each pair could be assumed to be equal by performing an F-test. The results of this analysis are provided in Table 3.

**Table 3:** The comparison of clearance and lane blockage depending on safety service patrol.

MOE^a^		Clearance Time						Lane Blockage Time					
Collision Type		1 Vehicle		2 Vehicles		3+ Vehicles		1 Vehicle		2 Vehicles		3+ Vehicles	
Safety Service Patrol		With	W/O**	With	W/O	With	W/O	With	W/O	With	W/O	With	W/O
Primary Incident	Number of Samples	9	139	27	170	15	107	9	139	27	170	15	107
	Average Duration (min)	48.6	88.5	48.6	96.2	53.1	81.9	24.2	38.2	14.1	49.7	30.3	57.6
	*P*-value (F-test)	2.018 $\times 10^{-5}$		2.2 $\times 10^{-16}$		1.349 $\times 10^{-4}$		0.048		2.726 $\times 10^{-13}$		0.001	
	Estimate Method	Welch											
	DOF***	60.3		194.0		51.8		11.7		184.0		42.3	
	T-statistic	−2.58		−3.18		−3.49		−1.15		−3.96		−2.84	
	*P*-value (T-test)	0.0124		0.0017		0.0010		0.2740		0.0001		0.0094	
Secondary Incident	Number of Samples	9	96	69	298	13	97	9	96	69	298	13	97
	Average Duration (min)	19.2	88.2	34.0	48.2	47.3	55.9	0	52.2	13.9	19.4	23.7	29.4
	*P*-value (F-test)	1.884 $\times 10^{-10}$		1. $411\times 10^{-7}$		0.3368		–		2. $726\times 10^{-5}$		0.1766	
	Estimate Method	Welch		Welch		Pooled Variance		–		Welch		Pooled Variance	
	DOF	98.1		181		108		95		155		108	
	T-statistic	−2.14		−3.20		−0.597		–		−1.23		−0.477	
	*P*-value (T-test)	0.0347		0.0016		0.552		–		0.219		0.634	

a MOE: Measure of Effectiveness **W/O: Without ***Degree of Freedom.

As indicated in Table 3, variances can be assumed to be statistically identical according to the confidence level, 0.95, for the case of three or more vehicle collisions in primary and secondary incidents. In these cases, a *t*-test was performed, assuming that they were subject to pooled variance. On the contrary, the degree of freedom (DOF) was assumed using the Welch method when the result of the f test rejected the null hypothesis of no difference between the variances of the groups. As a result, when the SSP program was involved in handling primary and secondary incidents, it was confirmed that lane blocking and incident clearance time was reduced. In particular, the results of the *t*-test indicated that the aid of the SSP program is associated with a statistically significant reduction in the lane blockage and incident clearance times for all types of vehicle collisions, except for the lane blockage time of a single vehicle crash in primary incidents.

### 5.2 Probability of secondary incidents

Survival analysis, correlation analysis, and binary logistic regression were performed to develop a predictive model for the probability of secondary incidents and to evaluate the relationships between predictors and the probability of secondary incident occurrence. First, the survival analysis was performed for the shoulder and lane(s) blocks, respectively. In this analysis, the survival rate is the percentage of primary incident that remains at that time and has not caused a secondary incident. For example, a survival rate of 80% means that 80% of incidents that have not been cleared at that time have not had a secondary incident. In contrast, 20% of incidents have had secondary incidents occur when the primary incidents last until that time. In another way, the survival rate is the probability of not having a secondary incident, since the primary event has not been cleared by that time.

To solve the issue of imbalance in the number of samples between primary incidents and general incidents, a data set was constructed randomly by selecting as many general incident samples as twice the number of primary incidents. The time taken from a primary incident to a secondary incident was calculated as the difference between the reported time of a primary incident and the recorded time of a secondary incident that occurred due to the primary incident. The probability of secondary incident occurrence increased as time increased after the initiation of the primary incident in shoulder and lane(s) blockage. Furthermore, survival analysis confirmed that 99.9% of the secondary incidents occurred within 4 h of the start of each primary incident. Consequently, the data processing threshold to analyze the exact correlation between the characteristics of the secondary incidents was set at 4 h. Cases where incident clearance time exceeded 4 h were removed as outliers.

**Fig. 2:**
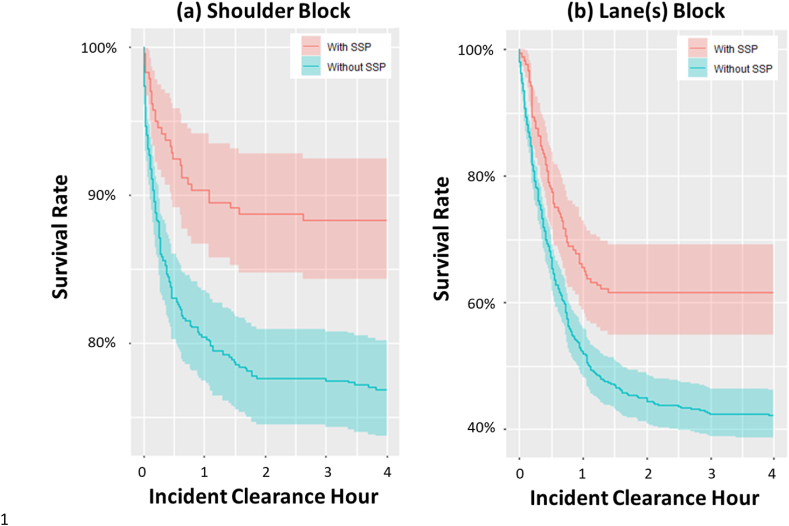
The results of the survival analysis. (a) Shoulder block; (b) Lane(s) block.

The survival rate, which represents the probability that a secondary incident does not occur, can be interpreted as statistically higher when the SSP is involved in dealing with an incident than the counterpart. Furthermore, it can be found that the interval where the survival rate becomes constant appears in a shorter incident clearance hour with the help of the SSP. This indicates that the SSP's aid can reduce the probability of secondary incident occurrences that would have occurred if the SSP had not intervened (Fig. 2a and b). Survival analysis showed that secondary incidents occurred within a short period of time from a primary incident, so appropriate countermeasures are necessary to quickly deal with primary incidents.

After data cleaning, correlation analysis was performed to determine the relationship between independent variables and the occurrence of secondary crashes. As a result, variables such as incident duration, injuries, fatality, number of cars involved in a case, indicator of the SSP program, and duration of lane blockage were found to have a statistically significant relationship with the occurrence of primary incidents. However, the correlation coefficient between incident clearance hour and the duration of the lane blockage was statistically significant at 0.41, so a multicollinearity issue was expected. Therefore, the duration of the incident was determined as representative of the time variables because its correlation coefficient with the dependent variable was higher than the duration of the lane blockage. Finally, five explanatory variables of the binary logit model were selected. Duration of the incident, number of injuries and cars, lane blocking indicator, and SSP program indicator.

In the binary logistic regression model, the total duration of the incident, blocking the lane due to an incident, and the number of injuries and involvement of the car were selected as independent variables. As discussed above, a stratified sampling method was applied to solve the imbalance problem between primary and general incidents. Since the number of primary incident samples remaining after data cleaning was 539, 1,078 general incidents (twice the number of primary incident samples). Therefore, a data set with a total of 1,617 cases was constructed. Finally, the data set was divided into a training set at 75% for model development and a test set at 25% for model validation. The resulting binary logit regression model is provided in Table 4.

**Table 4:** Summary of the binary logistic regression model.

Variables	Estimate Coefficients (β_i_)	Standard Error	Odds Ratio (exp (β_i_))	Average Marginal Effects	Z-Value	*P*-value
(Intercept)	−1.032	0.156	0.356	–	−6.620	3.58 $\times 10^{-11***}$
Incident Clearance Time	0.288	0.080	1.334	0.044	3.598	3.21 $\times 10^{-4***}$
Injuries	0.667	0.270	1.978	0.101	2.469	0.02*
Cars	1.168	0.152	3.214	0.177	−9.188	<2.00 $\times 10^{-16***}$
Only Shoulder Blocking (Y)	−1.397	0.099	0.247	−0.231	11.678	<2.00 $\times 10^{-16***}$
Safety Service Patrol (Y)	−0.785	0.192	0.456	−0.115	−4.089	$4.34\times 10^{-5***}$

Null deviance: 1,542.9 on 1,211° of freedom.

Residual deviance: 1,130.5 on 1,206° of freedom.

AIC: 1,142.5/Accuracy: 0.78/Sensitivity: 0.86/Specificity: 0.65.

Significant Level Codes: 0 ‘***’ 0.001 ‘**’ 0.01 ‘*’ 0.05.

From the binary logit results, it was confirmed that incident clearance time, injuries, and cars had statistically significant associations with the probability of secondary crash occurrence. The exponential value of the incident clearance time coefficient was 1.33, which indicates that the odds of secondary incidents increase by 33% as the duration of the incident increases by 1 h. Furthermore, the exponential values of the number of injuries and vehicles were analyzed at 1.98 and 3.21, indicating that the odds of secondary crashes occurring increase by 98% when the number of injuries increases by one person and 221% when the number of vehicles involved in an incident increases by one vehicle. On the other hand, for only shoulder blocking incidents, the coefficient was −1.397, indicating that the odds of secondary incident occurrence decrease by 75% compared to incidents with lane blocking. Furthermore, the odds of secondary incidents occurring when the SSP program was involved were 55% lower than when the SSP program was not deployed or used. Taking into account variables such as the number of injuries, the number of vehicles involved in an incident, shoulder blocking, and the SSP program, it is identified that the probability of secondary incident occurrence is not only affected by the duration of an incident, but also by the severity of the incident shown in Table 4. Additionally, it could be shown that SSP intervention significantly reduced secondary incident occurrence by reducing incident clearance time. The formula of the binary logistic regression model is presented in Equation (4).

(4) $$P=\frac{e^{-1.032+0.288*ICT+0.667*IJ+1.168*C-1.397*SBI\left(Y\right)-0.785*SSPI\left(Y\right)}}{1+e^{-1.032+0.288*ICT+0.667*IJ+1.168*C-1.397*SBI\left(Y\right)-0.785*SSPI\left(Y\right)}}\text{,}$$

Finally, the performance of the binary logit model was validated on the basis of sensitivity, specificity, and general accuracy. The remaining 405 incidents were randomly selected as the sample from the test set, 156 cases of primary incidents, and 249 cases of general incidents. For the validation analysis using test data, the sensitivity of the binary logistic regression model was 86%, the specificity was 65%, and the overall prediction accuracy of the model was 78%, which validates the performance of the estimated binary logistic regression model.

### 5.3 Spatial analysis of safety service patrol program

**Fig. 3:**
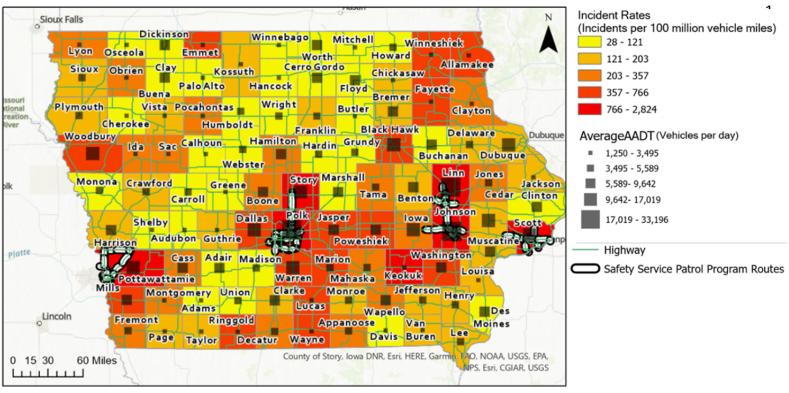
Incident rate by county in Iowa.

The objective of this part is to determine whether the coverage of the SSP program was properly planned from a traffic safety perspective by comparing the program routes with the regions where primary and secondary incidents occurred. First, the total incidents that occurred during the study period were aggregated by a county in Iowa using GIS software. Based on the total incident rates, it was found that the seven counties with the highest incident rates were: Polk, Scott, Pottawattamie, Johnson, Keokuk, Linn, and Story. The SSP program is currently in operation in each of these counties with the exception of Keokuk. However, the average AADT of all highway segments within Keokuk County was 1,697 vehs/day, which is only 8% of the average AADT of 20,535 vehs/day from the other top seven counties. This indicates that the regions where the incidents rate is high and the traffic volume is heavily concentrated are covered by the current SSP program. Fig. 3 provides a map that compares the incident rates for all Iowa counties.

**Fig. 4:**
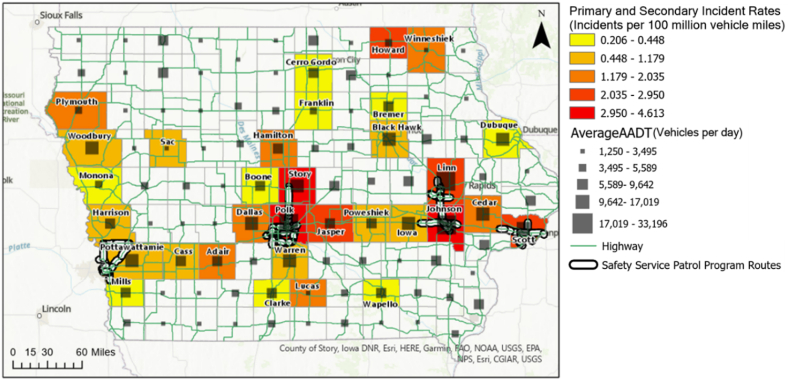
Primary and secondary incident rate by county in Iowa.

For the final stage of the spatial analysis, the incident rate for each county in Iowa was calculated by limiting the range of incidents to primary and secondary incidents, which show a high level of incident severity. The seven leading counties based on incident rates included Polk, Story, Johnson, Howard, Linn, Jasper, and Scott, as shown in Fig. 4. The program managed five of these counties, all except Howard and Jasper. Howard and Jasper ranked high in Iowa, considering the primary and secondary incident rates. However, Howard County's incident rate was the fourth highest in the state at 2.95, but only had four total primary and secondary incidents. Furthermore, the average AADT of the highway segment was 3,767 vehs/day, which was one-fifth of the other six counties 19,582 vehs/day. However, Jasper County had 38 primary and secondary incidents cases during the study period, more than three times higher than the Iowa average (12.3 cases on average). Furthermore, the average AADT for highway segments in Jasper County, 8,854 vehicles/day, was higher than the average AADT for Iowa (5,948 vehicles/day). However, there was no route covered by the SSP program in Jasper. Therefore, based on traffic safety, the spatial analysis indicated that Jasper should be considered a high priority when looking for the next county to apply the SSP program.

## 6 Conclusions

Primary and secondary incidents in Iowa account for more than 80% of vehicle collisions, and injuries and fatalities per 10,000 incidents are at least seven times higher than general incidents. Iowa DOT operates an SSP program in major metropolitan areas to expedite incident management and response. However, previous studies that analyze the advantages of the SSP program based on a detailed analysis of primary and secondary incidents were insufficient. Therefore, a comprehensive analysis of primary and secondary incidents was conducted that included the factors of primary incidents associated with secondary incidents, and whether the SSP program is properly operated based on traffic safety in Iowa. Consequently, the parameters for this study were derived by reviewing the existing literature and considering the limitations of existing data sources. This article was divided into a statistical analysis part for primary and secondary incidents and a spatial analysis part with regard to the SSP program to discern insights encompassing both incidents and the SSP program.

The SSP program was found to have the benefit of reducing incident clearance and lane blockage times among primary and secondary incidents. Furthermore, survival analyzes confirmed that SSP intervention in one incident could reduce the probability of a secondary incident and was helpful in prompt clearing up a primary incident. However, 99.9% of the secondary incidents occurred within 4 h of the primary incident. This could be interpreted to mean that the probability of secondary incident occurrence decreases when the duration of the primary incident is too long. This could be associated with reduced traffic speeds due to the incident clearance process, drivers being cautious, improved traffic, and improved information dissemination to incident drivers when agencies know that the clearance time will not be short. Furthermore, incident clearance time, injuries, cars, shoulder blocking indicator, and SSP indicator were selected as independent variables of the binary logistic regression model. Incident clearance time, injuries, and cars were confirmed to increase the likelihood of secondary incidents. Interpreting the BLR model confirmed that the longer the incident processing time and the more severe the incident, the higher the probability of occurrence of a secondary incident.

The results of the spatial analysis of the SSP program indicated that the coverage area of the SSP program currently operating in the main areas of Iowa is appropriate from the point of view of traffic safety. It also indicated that Jasper should be considered a priority when expanding the program in the future, considering the primary and secondary incident rates and the overall volume of traffic.

The significance of this research is that the evaluation of Iowa DOT's SSP program was conducted from a macro-perspective based on traffic safety. It provided consequential insights that statistical analyzes of primary and secondary incidents, key elements in incident management, and organized the characteristics of the incidents systematically and comprehensively. Additionally, the integrated analysis method and approach proposed in this study are expected to be useful for local governments and other states or regional transportation agencies with incident data capable of identifying secondary incidents, traffic volume data, and SSP route data to follow a similar procedure when analyzing the safety benefit of a program. Finally, the findings can be valuable to transportation authorities, policymakers, and practitioners to improve incident management strategies and implement more effective SSP programs by providing beneficial evidence obtained through the application of the program on site.

The considerations for future research, based on the data, analysis and findings, include the following: consideration of more detailed incident types and improvements in spatial analysis. For this research, various types of incidents were aggregated into a single category when comparing the severity of incidents, so it was not possible to determine differences between different types of events. For example, information related to whether the incident was due to a vehicle collision or if it was due to stalled vehicles was not available. Therefore, it is necessary to analyze the severities and likelihood of secondary incidents by incident types in future research. Next, the transferability of the binary logistic regression model presented in this study should be checked using data from other geographical areas in addition to Iowa before directly using the estimation model in new areas. Furthermore, when performing spatial analysis, the appropriateness of the routes assigned to the SSP program was evaluated only on the basis of the incident rate. However, since the SSP program requires a budget, a benefit-cost analysis could be incorporated to capture the cost constraints agencies face. Furthermore, incident rate comparison could be improved in future studies by increasing the sample size and incorporating exposure in count regression models to capture potentially non-linear relationships between exposure and number of incidents.

## Author contribution statement

Minsoo Oh, Jonathan Wood: Conceived and designed the analysis; Analyzed and interpreted the data; Contributed analysis tools or data; Wrote the paper.

Jing Dong-O’Brien: Conceived and designed the analysis; Contributed analysis tools or data; Wrote the paper.

## Data availability statement

The authors do not have permission to share data.

## Declaration of competing interest

The authors declare that they have no known competing financial interests or personal relationships that could have appeared to influence the work reported in this paper.
